# Carvacrol exhibits potent anti-*Klebsiella* activity, whereas the multicomponent composition of essential oils may contribute to their enhanced antibacterial activity

**DOI:** 10.3389/fphar.2026.1918941

**Published:** 2026-09-18

**Authors:** Magaly Jaquelyne Lara-Garcia, Luz Ofelia Franco-Sandoval, Axel David Briones-Guzmán, Mariana Romo-Castillo

**Affiliations:** 1 Facultad de Ciencias de la Salud, Universidad Autónoma de Tlaxcala, Tlaxcala, Mexico; 2 Laboratorio de Investigación en Patógenos Respiratorios, Hospital Infantil de México Federico Gómez, Ciudad de México, Mexico; 3 Laboratorio de Investigación en Enfermedades Infecciosas y Parasitarias, Hospital Infantil de México Federico Gómez, Ciudad de México, Mexico; 4 Investigadores por México SECIHTI, Secretaría de Ciencia, Humanidades, Tecnología e Innovación, Ciudad de México, Mexico

**Keywords:** carvacrol, essential oil, *Klebsiella*, oregano, summer savory, thyme

## Abstract

Multidrug-resistant (MDR) *Klebsiella* infections represent an increasing therapeutic challenge, underscoring the need for complementary antimicrobial strategies. Essential oils (EOs) are complex mixtures of bioactive compounds, and their antibacterial effects may not be fully explained by their predominant constituents. We compared the antibacterial activity of isolated carvacrol with that of oregano (*Origanum vulgare*), summer savory (*Satureja hortensis*), and thyme (*Thymus vulgaris*) essential oils against MDR *Klebsiella* isolates and evaluated their ability to enhance conventional antibiotics. Essential oils were chemically characterized by gas chromatography–mass spectrometry (GC–MS). Antibacterial activity was evaluated by agar diffusion, minimum inhibitory concentration (MIC), and minimum bactericidal concentration (MBC) assays. Interactions with ceftazidime, gentamicin, and ciprofloxacin were assessed using full checkerboard assays and fractional inhibitory concentration indices (FICIs). Toxicity was evaluated in *Galleria mellonella* and *Tenebrio molitor* larvae. GC–MS identified carvacrol as the predominant constituent of oregano (71.47%) and summer savory (48.83%) oils, whereas thyme oil contained low levels of carvacrol (1.83%). Whole oregano and summer savory oils exhibited greater bactericidal activity than isolated carvacrol in several comparisons, despite the high carvacrol content of these oils. Thyme oil also showed substantial antibacterial activity despite its low carvacrol concentration, indicating that activity was not determined solely by carvacrol abundance. Toxicity assays indicated low toxicity for carvacrol, oregano, and thyme oils under the tested conditions, whereas summer savory showed moderate, species-dependent toxicity. Overall, whole essential oils exhibited antibacterial activities that could not be explained solely by their carvacrol content, supporting a contribution of their multicomponent phytochemical composition. Their ability to potentiate conventional antibiotics further supports their potential as complementary antimicrobial agents against MDR *Klebsiella*. Further studies using purified constituents and defined combinations are warranted to determine the specific mechanisms underlying these effects.

## 1 Introduction

*Klebsiella* is a well-known opportunistic pathogen that primarily affects immunocompromised patients. It is a Gram-negative, anaerobic, rod-shaped enterobacterium (Dong et al., 2022). It is widely distributed and frequently commensal in the human digestive tract and nasopharynx, and it is also found in soil, water, and animal feces. Two principal species with nosocomial impact are *Klebsiella pneumoniae* and *Klebsiella oxytoca* (Kijineh et al., 2024). In children, *Klebsiella* infections can present as pneumonia, urinary tract infections (UTIs), neonatal sepsis, and, in more severe cases, meningitis (Atay Ünal et al., 2026). One of the most concerning aspects of *Klebsiella* infections in pediatric patients is the increasing prevalence of antibiotic resistance (Wyres and Holt, 2016; Atay Ünal et al., 2026). Both *K. oxytoca* and *K. pneumoniae* have demonstrated significant resistance to multiple antibiotic classes, complicating treatment and posing a major threat to public health (Asokan et al., 2025). Managing *Klebsiella* infections in children is increasingly challenging because conventional antibiotics are often less effective against resistant strains (Palacios-Baena et al., 2021). As a result, the search for new antibiotics and alternative therapies has become critical. Given the limitations of synthetic antibiotics, which often raise safety concerns and foster resistance, natural compounds offer a compelling alternative for combating these increasingly resilient pathogens.

Historically, plants have been used in various medicinal preparations to treat many illnesses and infections (Alexandrova et al., 2026; Guan et al., 2026; Mkhabela et al., 2026; Saraswathi et al., 2026; Šemeláková et al., 2026). These practices employ whole extracts, infusions, decoctions, or essential oils (EOs) containing hundreds of chemical constituents. However, modern pharmaceutical development has largely favored isolating individual bioactive molecules (Perreault et al., 2026). This reductionist approach assumes that therapeutic activity can be attributed primarily to a single molecule, but increasing evidence suggests that antibacterial activity often arises from interactions among multiple constituents within a phytochemical matrix. Interactions among plant metabolites operate at several pharmacological levels (Caesar and Cech, 2019): pharmacodynamic synergy (several molecules interacting with different targets or pathways), pharmacokinetic synergy (some components increasing absorption, distribution, metabolism, or bioavailability of other components), protective synergy (molecules that stabilize unstable compounds and minimize negative consequences), and resistance (compounds that block bacterial resistance systems, such as efflux pumps or enzymes, thereby keeping the principal molecule active). The modern drug discovery focus on isolating, purifying, and standardizing a single molecule fails to capture the effect of the whole-plant matrix (Tamboli, 2026). The aim of this study was to compare the activity of isolated components with complete essential oils and determine whether the reductionist approach is effective and offers an advantage over whole-plant extracts. Essential oils contain dozens to hundreds of volatile compounds, and many of them have been identified as major contributors to antibacterial activity, including carvacrol, thymol, eugenol, cinnamaldehyde, terpinen-4-ol, linalool, and α-pinene (Hajian-Maleki et al., 2025; Kashi et al., 2025; Kot et al., 2026; Magdy et al., 2026; Zhao et al., 2026).

Carvacrol is highly concentrated in the essential oils of the Lamiaceae family, including *Origanum* sp., *Thymus* sp., *Satureja* sp., *Zataria* sp., *Ocimum* sp., and *Mentha* sp. Beyond direct cellular disruption, carvacrol has been shown to interfere with bacterial quorum sensing and inhibit biofilm formation, both of which are critical virulence factors for many pathogens. Moreover, carvacrol’s efficacy extends to combating drug-resistant bacteria, suggesting its potential as an adjuvant to conventional antibiotics (Li et al., 2022; Asadi et al., 2023). Carvacrol is a naturally occurring monoterpenoid phenolic compound predominantly found in essential oils such as oregano, summer savory, and thyme. In this study, we analyze carvacrol and compare its activity with three other essential oils. The aim of the study is to determine whether a reductionist approach is better than using full plant extracts.

## 2 Materials and methods

### 2.1 Compounds and essential oils

Citronellol (95%, CAS: 106-22-9, Sigma-Aldrich Cat. W230901), limonene (97%, CAS: 5989-27-5, Sigma-Aldrich Cat. 193164), beta-pinene (CAS: 19902.08-0, Sigma-Aldrich Cat. 80607), eucalyptol (CAS: 470-82-6, Sigma-Aldrich Cat. 29210), linalool (97%, CAS: 78-70-6, Sigma-Aldrich Cat. L2602), carvacrol (99%, CAS: 488-75-2, Sigma-Aldrich Cat. W224511), linalyl acetate (CAS: 115-95-7, Sigma-Aldrich Cat. W263613), geranyl acetate (>97%, CAS: 105-87-3, Sigma-Aldrich Cat. 173495), and gamma-terpinene (>97%, CAS: 99-85-4, Sigma-Aldrich Cat. 223190) were obtained from Merck.

Essential oils of oregano (*Origanum vulgare*), summer savory (*Satureja hortensis*), and thyme (*Thymus vulgaris*) were obtained from Hela Productos, a local producer in Coyoacán, Mexico City, Mexico. They cultivated the plants organically without pesticides or synthetic fertilizers. The aerial parts were harvested before flowering and dried in the shade outdoors. The essential oils were extracted by steam distillation and stored in amber bottles in a cool place. After extraction, the oils were analyzed by gas chromatography (GC) using an Agilent 7890N coupled to an Agilent Technologies 5977A mass spectrometer (GC–MS). The essential oil constituents were characterized by matching their mass spectra to the NIST (National Institute of Standards and Technology, U.S. Department of Commerce, Gaithersburg, MD, United States) compound library (Supplementary Tables S1–S3). To standardize the assays and the composition of the oils, we used only essential oils obtained from the same collection and a single distillation process.

### 2.2 Strains and culture conditions

*Clinical Klebsiella* strains were obtained from the strain collection of the Clinical Laboratory at the Hospital Infantil de México Federico Gómez. The genus and species of the strains were determined using the VITEK-2 Compact System. Species identification was based on biochemical profiles generated by the GN identification cards, including differentiation of *K. pneumoniae* and *K. oxytoca*. When required, species identification was confirmed by conventional biochemical testing, including indole production, with *K. pneumoniae* being indole-negative and *K. oxytoca* indole-positive. Antibiotic susceptibility testing was performed using the Kirby–Bauer method with ciprofloxacin (CIP, 5 µg), ceftazidime (CAZ, 30 µg), gentamicin (GEN, 10 µg), nitrofurantoin (NFT, 300 µg), and tigecycline (TGC, 15 µg), using the reference Gram-negative strain *K. pneumoniae* ATCC 700603. Strains were defined as multidrug-resistant (MDR) when they were resistant to at least one agent in three or more antimicrobial categories, according to the standardized international definition (Magiorakos et al., 2012). Three MDR *K. pneumoniae* and three MDR *K. oxytoca* strains were used in this study (Supplementary Table S4). The strains were cultured in BHI broth or on standard blood agar at 37 °C. All strains were stored in 10% glycerol and 10% nonfat milk at −80 °C for subsequent analysis.

Each clinical isolate was treated as an independent biological replicate (experimental unit). Technical triplicates were performed solely to verify reproducibility and were averaged before data analysis. Technical replicates were not treated as independent observations for statistical testing.

### 2.3 Antibacterial activity of compounds and essential oils

Antibacterial activity was assessed using the disk diffusion method on Mueller–Hinton agar (MHA). Each strain was cultured in 5 mL of Mueller–Hinton broth at 35 °C for 18 h with vigorous agitation. The culture was then diluted to 0.5 McFarland, and 100 μL was used to inoculate and spread on MHA. Blank standard disks were impregnated with 10 μL of compounds (300 μg/mL) or essential oils (50% v/v). Concentrations expressed as percentages (v/v) were converted to mg/mL using the density of each compound or essential oil, using the following equation:

$$Concentration\;\left(\frac{mg}{mL}\right)=\left(\frac{\%\;v/v}{100}\cdot density\;\left(\frac{g}{mL}\right)\right)\cdot 1000.$$

The densities were 0.976 g/mL for carvacrol, 0.910 g/mL for oregano, 0.920 g/mL for summer savory, and 0.900 g/mL for thyme. The carvacrol concentration in each oil was then calculated based on its percentage composition.

Minimum inhibitory concentration (MIC) and minimum bactericidal concentration (MBC) were determined by microdilution, as previously reported (Romo-Castillo et al., 2023). Overnight bacterial cultures were adjusted to a 0.5 McFarland standard and diluted in Mueller–Hinton broth to achieve a final bacterial inoculum of approximately 5 × 10^5^ CFU/mL in each assay well. Serial two-fold dilutions of each antimicrobial agent were prepared in DMSO or fractionated coconut oil, and the final volume of each well was 200 μL. Following incubation at 37 °C for 18 h, bacterial growth was assessed by visual inspection and confirmed using a resazurin reduction assay. MIC and MBC of antibiotics were determined using concentrations ranging from 0.25 to 256 μg/mL for CAZ and GEN and from 0.03 to 32 μg/mL for CIP, in accordance with the CLSI protocol. A concentration range of 50% to 0.25% (v/v) was employed for carvacrol and essential oils. DMSO and fractionated coconut oil served as controls. For resazurin reduction analysis, 10 µL of 0.015% resazurin was added to each well, and the mixture was incubated for 2 h at room temperature to observe the color change (Saeed et al., 2021). The MIC was defined as the lowest concentration that completely inhibited visible bacterial growth, with resazurin retaining its natural color (blue/purple). For MBC determination, 5 µL from each well was plated onto MHA and incubated for 24 h at 37 °C. The MBC was defined as the lowest concentration that resulted in no detectable colony growth following subculture. Although no apparent interference with resazurin color development was observed during the assays (not shown), we recognize that highly hydrophobic compounds may affect optical interpretation. Therefore, MIC determinations were confirmed by visual assessment, and MBC determinations were performed through bacterial subculture.

### 2.4 Potentiation and synergistic effect between compound/EO and antibiotics

The potential synergistic effect between the compound/EO and antibiotics was assessed using a standard checkerboard assay with the compound/EO and a gradient of each antibiotic, as described previously. Antibiotics were evaluated at concentrations ranging from 0.062 to 256 μg/mL for CAZ and GEN and from 0.007 to 32 μg/mL for CIP. EOs were evaluated at concentrations ranging from 50% to 0.01%, corresponding to 488–0.119 mg/mL for carvacrol, 455–0.111 mg/mL for oregano, 460–0.112 mg/mL for summer savory, and 450–0.110 mg/mL for thyme. The fractional inhibition concentration (FIC) for each drug was calculated by dividing the MIC of the combination by the MIC of the drug alone. The fractional inhibition concentration index (FICI) was calculated as the sum of the FICs of each drug. FICI values were recorded as synergy (FICI <0.5), additive (FICI = 0.5–1.0), indifferent (FICI = 1.0–4.0), or antagonism (FICI >4). Assays were performed in triplicate across independent experiments.

A modified checkerboard assay was also performed using a fixed subinhibitory concentration of carvacrol/EO (1/4 MIC) combined with serial two-fold dilutions of the selected antibiotic. The antibiotic MIC in the presence of the natural product was compared with the MIC of the antibiotic alone. Results were expressed as the fold reduction in antibiotic MIC and were interpreted as evidence of antibiotic potentiation. Because the carvacrol/EO concentration was fixed at 1/4 MIC rather than systematically varied with the antibiotic, this assay was not considered a conventional two-dimensional checkerboard assay. Therefore, it was not used as definitive evidence of pharmacological synergy but was useful for analyzing the potentiation of antibiotic activity.

### 2.5 Toxicity of the compound/EO

In this study, *G. mellonella* and *Tenebrio molitor* larvae were used as model organisms to assess drug toxicity. *G. mellonella* larvae were donated by the Microbiology and Parasitology Department, Faculty of Medicine, UNAM. The larvae were kept in plastic pots at 28 °C and reared on an artificial diet of wheat, oats, glycerin, and honey. *G. mellonella* larvae measuring 2–3 cm and weighing 180–300 mg, with light color and no melanization, were selected for the assay. *T. molitor* larvae were obtained from commercial producers and kept in plastic boxes at room temperature. Their diet and substrate consisted of oat flakes, with fruits or vegetables (such as apples and carrots) occasionally added. *T. molitor* larvae weighing 90–130 mg were selected for the assay. Larvae were randomly assigned to treatment groups and kept in Petri dishes, in groups of 10, for 24 h before the assay. They were then treated with 10 µL of each component/EO, administered using a Hamilton syringe. Toxicity assays were performed using concentrations corresponding to twice the MIC determined for each compound. Because the MICs differed among treatments, the resulting concentrations were approximately 40% (v/v) for carvacrol and 25% (v/v) for EOs. After inoculation, the larvae were incubated at 37 °C for 24 and 48 h. The adapted *G. mellonella* health index score (Champion et al., 2018) and the *T. molitor* health index score (proposed in this study) were used to evaluate activity levels, cocoon formation, melanization, and survival (Table 1). The *T. molitor* score was based on predefined observations of larval mobility, responsiveness, melanization, and general appearance. It was used as a complementary exploratory measure rather than as a validated toxicity endpoint. The assays were performed in triplicate for statistical analysis.

**TABLE 1:** Insect health index score values used as reference to value toxicity of the compounds.

Category	Description	*T. molitor*	*G. mellonella*
Activity	No movement	0	0
	Minimal movement on stimulation	1	1
	Moved when stimulated	2	2
	Move without stimulation	3	3
Cocoon formation	No cocoon	NA*	0
	Partial cocoon	NA*	1
	Full cocoon	NA*	2
Melanization	Black larvae	1	0
	Black spots on brown larvae	2	1
	3 spots on larvae	3	2
	<3 spots on larvae	4	3
	No melanization	5	4
Survival	Dead	1	0
	Alive	2	1

* NA, not applicable.

### 2.6 Statistical analyses

Statistical analyses were conducted using GraphPad Prism 10, including the Shapiro–Wilk test. Because several datasets did not meet the assumption of normality and sample sizes were limited, non-parametric analyses were performed. Differences among treatments were evaluated using the Kruskal–Wallis test, followed by Dunn’s multiple-comparison test with a Bonferroni correction. Statistical significance was set at *p* < 0.05. Each clinical isolate was treated as an independent biological experimental unit. Technical replicates from the same isolate were used to assess experimental reproducibility and were averaged before statistical analysis; they were not considered independent observations. Because only three clinical isolates per species were included, the analyses were considered exploratory and were used primarily to support descriptive comparisons among experimental groups. MIC and MBC values, which represent discrete two-fold dilution endpoints, were evaluated primarily at the individual isolate level and are presented descriptively. Given the limited number of biological replicates, statistical results were interpreted cautiously.

## 3 Results

### 3.1 Antibacterial activity of natural compounds against *Klebsiella pneumoniae* and *Klebsiella oxytoca*

Research on natural antibacterial compounds often adopts a reductionist “specific component” approach. To confirm antibacterial activity against clinical MDR *Klebsiella* strains, nine compounds were evaluated using the disk diffusion method at 50% (v/v). The components are listed in Table 2, which summarizes their chemical characteristics and the essential oils in which they occur. Three MDR *K. pneumoniae* (Kpn) and three MDR *K. oxytoca* (Koxy) clinical strains isolated from pediatric patients at the Hospital Infantil de México Federico Gómez (HIMFG) were used. The Kirby–Bauer assay showed that, although citronellol and linalool exhibited antibacterial activity against these strains, only carvacrol demonstrated effective activity, as indicated by an inhibition zone >15 mm (Figure 1A). To compare the antibacterial efficacy of individual compounds with that of whole essential oils, three EOs with high, medium, and low carvacrol concentrations were selected. The selected essential oils were oregano, typically ranging from 50% to more than 80% carvacrol; summer savory, typically ranging from 30% to 50%; and thyme, ranging from 1% to 10%. The selection of *Origanum vulgare* (oregano), *S. hortensis* (summer savory), and *T. vulgaris* (thyme) was supported by both their phytochemical composition and their long-standing use in traditional medicine. These aromatic species, all members of the Lamiaceae family, have been widely used in traditional medical systems in the Mediterranean and Middle East to treat infectious diseases, including respiratory, gastrointestinal, oral, and skin infections. After chromatographic analysis, we identified the EOs used in this study as containing 71.47% (oregano), 48.83% (summer savory), and 1.83% (thyme) (Figures 1B–D; Supplementary Tables S1–S3). Based on GC–MS quantification, the equivalent carvacrol concentrations were estimated at 199.7 mg/mL for oregano oil, 136.2 mg/mL for summer savory oil, and 5.1 mg/mL for thyme oil.

**TABLE 2:** Compounds analyzed to identify an antibacterial activity against *Klebsiella* strains.

Compound	Structure	Chemical characteristics	Essential oils that contain it	References
Citronellol	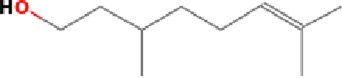	Monoterpenoid alcohol; C_10_H_20_O; rose-like scent. MW: 156.2652 g/mol	Citronella, rose, geranium, and lemongrass	Nakahara et al. (2013); Başer and Buchbauer (2020)
Limonene	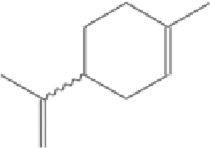	Monoterpene hydrocarbon; C_10_H_16_; citrus aroma. MW: 136.2340 g/mol	Orange, lemon, lime, grapefruit, and peppermint	Chiappisi and Grillo (2018); Başer and Buchbauer (2020)
Beta-pinene	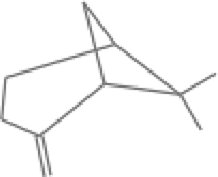	Monoterpene hydrocarbon; C_10_H_16_; pine scent. MW: 136.2340 g/mol	Pine, rosemary, basil, and parsley	Salehi et al. (2019); Başer and Buchbauer (2020)
Eucalyptol	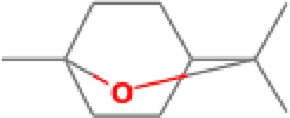	Monoterpene ether; C_10_H_18_O; minty, camphoraceous. MW: 154.2493 g/mol	Eucalyptus, bay laurel, rosemary, and sage	Başer and Buchbauer (2020); Moussa et al. (2024)
Linalool	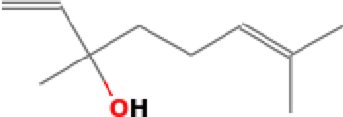	Monoterpene alcohol; C_10_H_18_O; floral scent. MW: 154.2493 g/mol	Lavender, basil, coriander, rosewood, and neroli	Pereira et al. (2018); Başer and Buchbauer (2020)
Carvacrol	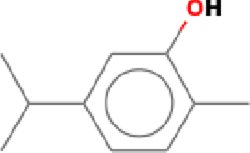	Monoterpenoid phenol; C_10_H_14_O; pungent odor. MW: 150.2176 g/mol	Oregano, thyme, and savory	Han et al. (2017); Başer and Buchbauer (2020); Magdy et al. (2026)
Linalyl acetate	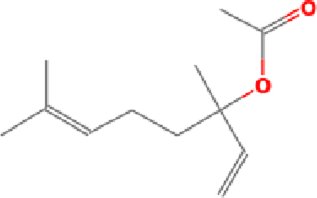	Monoterpenoid ester; C_12_H_20_O_2_; fruity aroma. MW: 196.2860 g/mol	Lavender, clary sage, bergamot, and petitgrain	Pereira et al. (2018); Başer and Buchbauer (2020)
Geranyl acetate	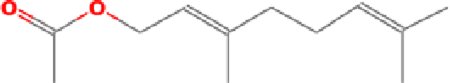	Monoterpenoid ester; C_12_H_20_O_2_; floral-fruity aroma. MW: 196.2860 g/mol	Palmarosa, citronella, lemongrass, and geranium	Başer and Buchbauer (2020); Jaramillo et al. (2024)
Gamma-terpinene	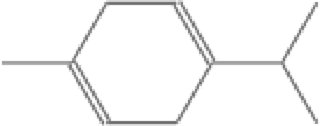	Monoterpene hydrocarbon; C_10_H_16_; citrusy-spicy odor. MW: 136.2340 g/mol	Tea tree, cumin, coriander, ginger, and lemon	Giweli et al. (2012); Başer and Buchbauer (2020)

**FIGURE 1:**
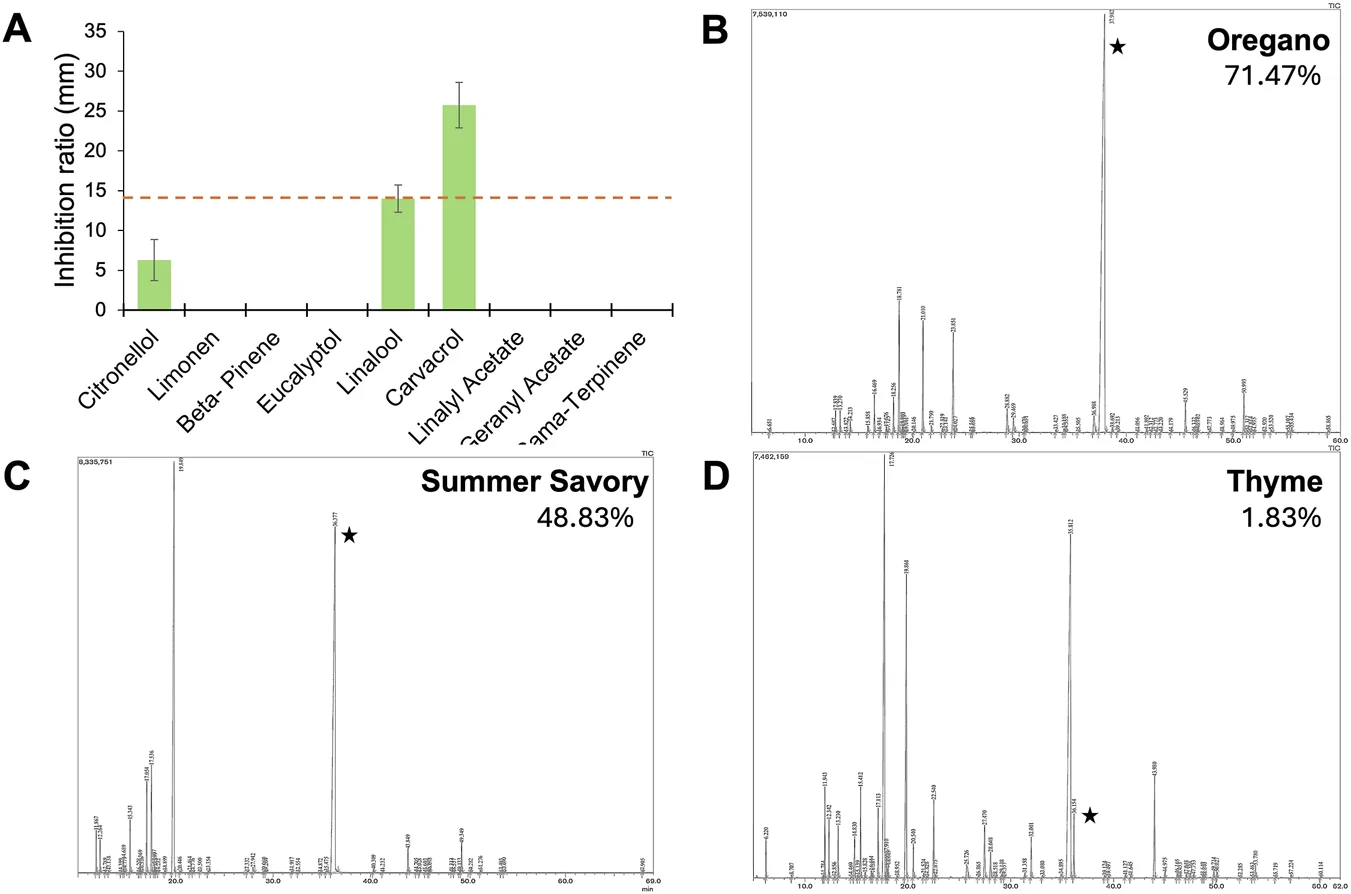
Antibacterial activity of natural compounds against clinical MDR *Klebsiella pneumoniae* (Kpn) and *Klebsiella oxytoca (Koxy)* strains. **(A)** Kirby–Bauer assay of compounds after 18 h of incubation at 37 °C. **(B)** Gas chromatogram of oregano essential oil. **(C)** Gas chromatogram of summer savory essential oil. **(D)** Gas chromatogram of thyme essential oil. The star indicates the carvacrol peak. Full information on the GC–MS chromatograms is provided in Supplementary Tables S1–S3.

### 3.2 MIC and MBC of carvacrol and essential oils

The MIC and MBC of the essential oils and carvacrol alone were determined using the microdilution method. The results indicate that essential oils exhibit greater antibacterial activity and are effective at lower concentrations than carvacrol alone (Figures 2A,C). The Kruskal–Wallis analysis of whole extracts showed significant differences among treatments (H = 20.0638, *p* = 0.0001646), and Dunn’s multiple-comparison test revealed significant differences between carvacrol/oregano (*p* = 0.00007583), carvacrol/summer savory (*p* = 0.005351), oregano/thyme (*p* = 0.01339), and summer savory/thyme (*p* = 04786) for *K. pneumoniae* (Figure 2A).

**FIGURE 2:**
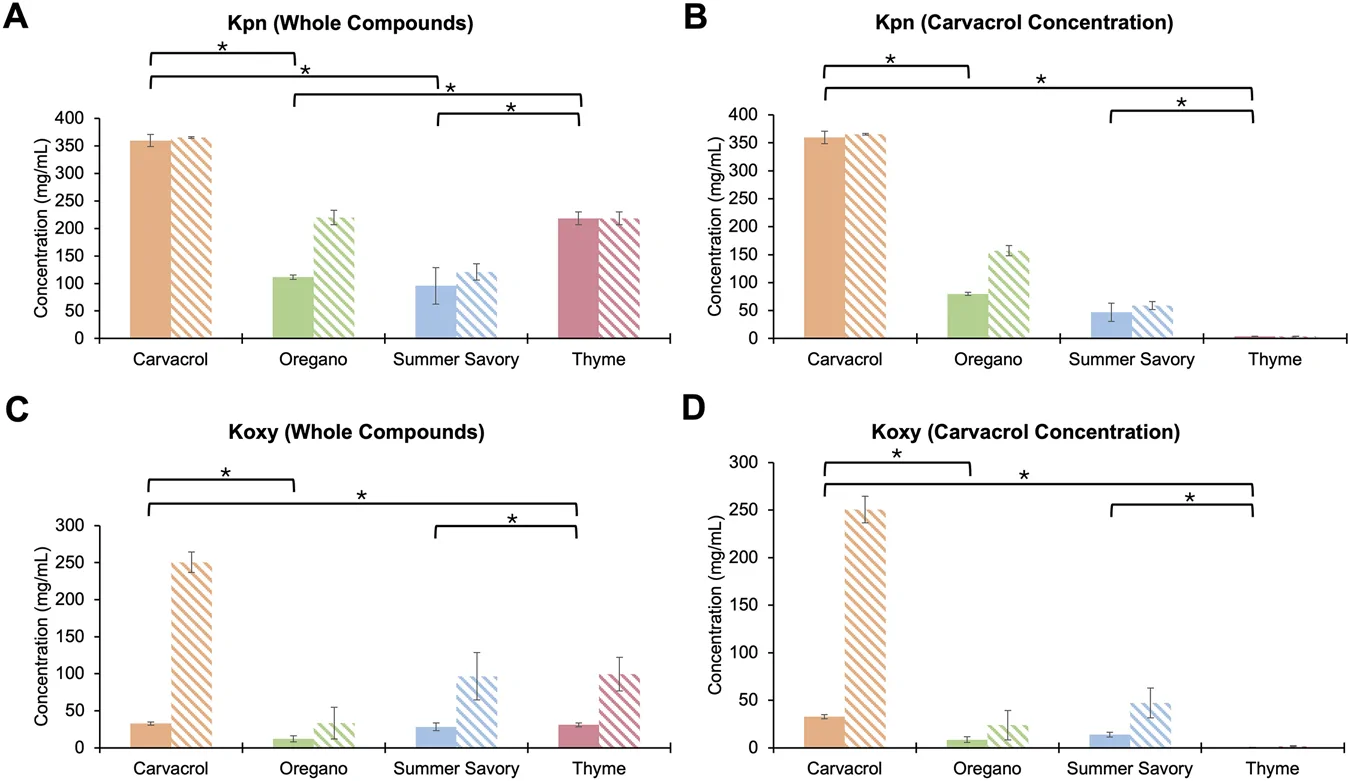
Minimum inhibitory concentration (MIC) and minimum bactericidal concentration (MBC) of carvacrol and essential oils (EOs) against clinical strains of *Klebsiella pneumoniae* and *Klebsiella oxytoca*. **p < 0.01. MIC and MBC based on whole extract concentrations from *K. pneumoniae* **(A)** and *K. oxytoca* **(C)** were compared with the carvacrol concentrations in each compound (**(B,D)**, respectively).

For *K. oxytoca* (Figure 2C), the Kruskal–Wallis analysis detected significant differences (H = 16.1432, *p* = 0.00106) among carvacrol/oregano (*p* = 0.00008038), oregano/summer savory (*p* = 0.02143), and oregano/thyme (*p* = 0.008568). When the carvacrol concentration of each essential oil was plotted (Figure 2B for *K. pneumoniae* and Figure 2D for *K. oxytoca*), the carvacrol concentrations in the oils were lower than those used for carvacrol alone. The Kruskal–Wallis analysis of whole extracts showed significant differences among treatments (H = 22.0212, *p* = 0.00006457) in *K. pneumoniae* (Figure 2B), and Dunn’s multiple-comparison test revealed significant differences between carvacrol/summer savory (*p* = 0.002998), carvacrol/thyme (*p* = 0.000008513), and oregano/thyme (*p* = 0.002998). For *K. oxytoca* (Figure 2D), the Kruskal–Wallis test detected a difference (H = 21.8662, *p* = 0.0000006955), and Dunn’s multiple-comparison test revealed differences between carvacrol/oregano (*p* = 0.003102), carvacrol/thyme (*p* = 0.00009157), and summer savory/thyme (*p* = 00.302). These results suggest that other molecules present in the oils exert a synergistic effect with carvacrol, enabling the use of lower concentrations to achieve optimal antibacterial activity. Although the MIC values expressed in mg/mL appear relatively high compared with those of purified conventional antibiotics, they are within the range commonly reported for complex essential oils. EOs are multicomponent mixtures in which only a fraction of the total mass corresponds to antibacterial constituents, and their hydrophobic nature often necessitates higher nominal concentrations to achieve homogeneous dispersion in aqueous media.

### 3.3 Antibiotic-potentiating and synergistic effect of carvacrol and essential oils

The antibiotic-potentiating effects of carvacrol and essential oils were then evaluated in the presence of CAZ, GEN, and CIP. To assess synergy, a classic checkerboard method was used (Figure 3), and the FICI was calculated. Most combinations showed synergistic interactions (FICI ≤ 0.5), whereas carvacrol–ciprofloxacin against *K. pneumoniae* (FICI = 0.55) and thyme essential oil–ciprofloxacin against *K. oxytoca* (FICI = 0.53) showed additive interactions.

**FIGURE 3:**
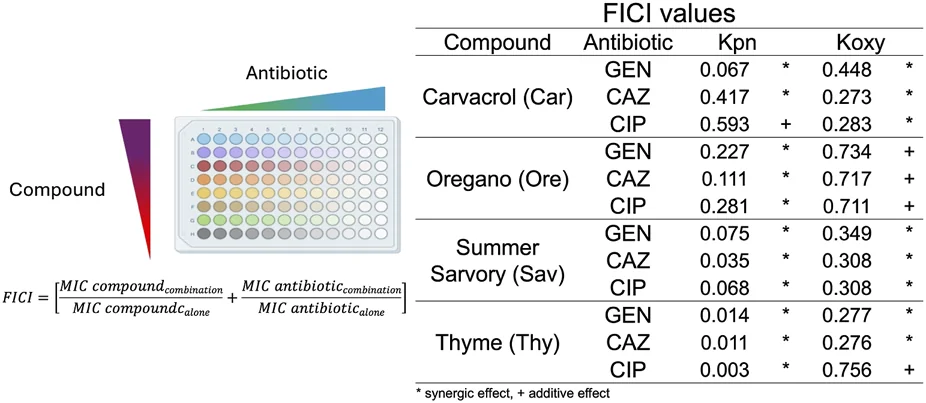
Fractional inhibitory concentration index (FICI) values from a checkerboard assay evaluating the antibiotic-potentiating effects of carvacrol and essential oils against multidrug-resistant *Klebsiella pneumoniae* and *Klebsiella* oxytoca. Carvacrol (Car), oregano essential oil (Ore), summer savory essential oil (Sav), and thyme essential oil (Thy) were evaluated for their effects on the antimicrobial activities of gentamicin (GEN), ceftazidime (CAZ), and ciprofloxacin (CIP).

Then, a modified checkerboard assay was performed using a constant subinhibitory concentration of the compounds (1/4 MIC) and a gradient of antibiotics (Figure 4). In *K. pneumoniae*, the Kruskal–Wallis analysis showed significant differences among GEN and GEN + treatments (H = 26.9819, *p* = 0.00002005), and Dunn’s multiple-comparison test revealed significant differences between GEN and GEN + Ore (*p* = 0.01563), GEN + Sav (*p* = 0.001292), and GEN + Thy (*p* = 0000491). For CAZ, the Kruskal–Wallis analysis (H = 16.8785, *p* = 0.002041) and Dunn’s multiple-comparison test showed significant differences when oregano (*p* = 0.005791), summer savory (*p* = 0.01909), and thyme (*p* = 0.0008342) were added. Finally, for CIP, differences among treatments were observed (H = 21.5388, *p* = 0.0002475), and Dunn’s multiple-comparison test revealed significant differences when summer savory (*p* = 0.001137) and thyme (*p* = 0.0001222) were added.

**FIGURE 4:**
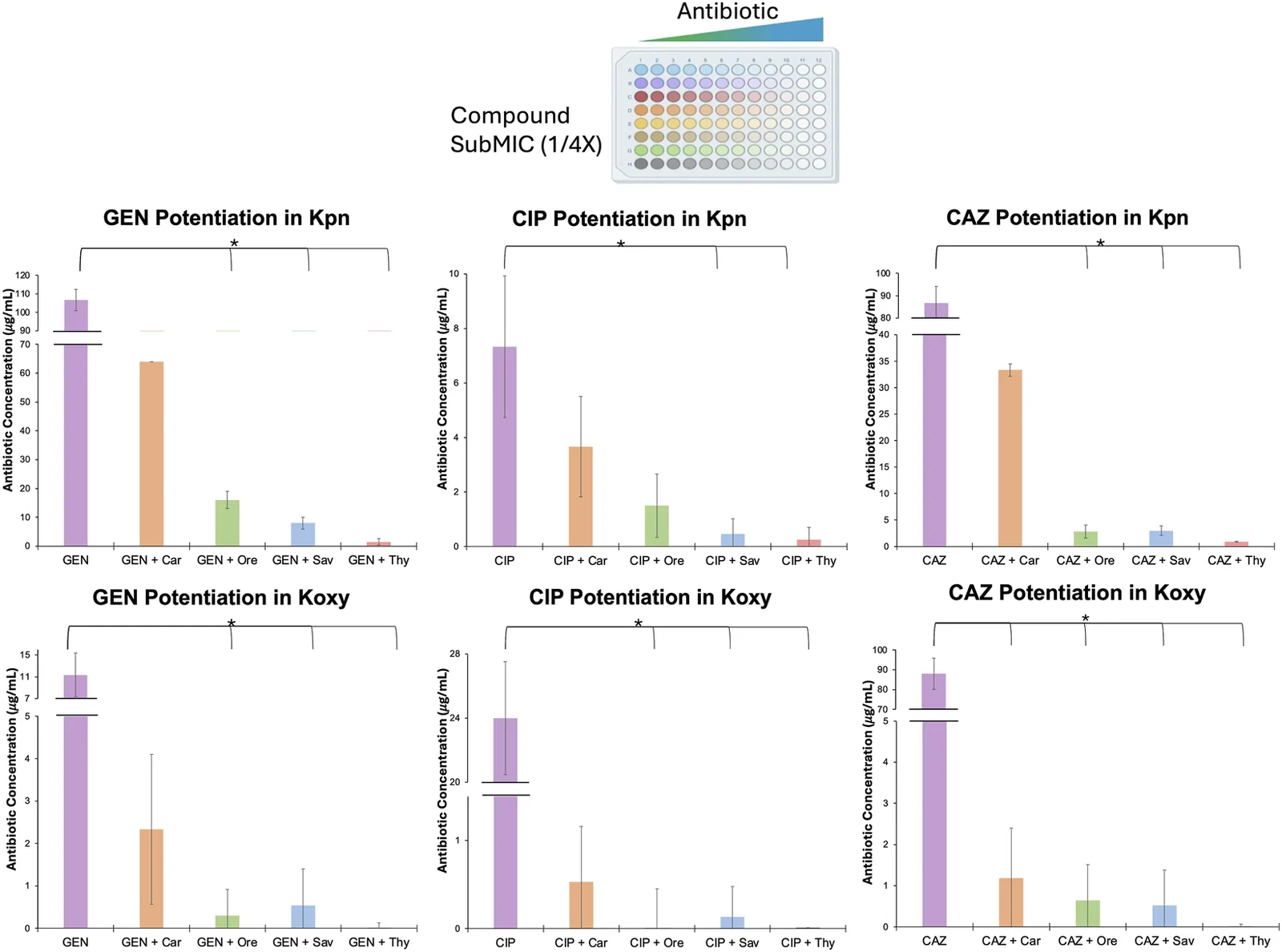
Antibiotic potentiation by carvacrol and essential oils. Modified checkerboard assay evaluating the effects of subinhibitory concentrations (1/4MIC) of carvacrol (Car), oregano essential oil (Ore), summer savory essential oil (Sav), and thyme essential oil (Thy) on the antimicrobial activities of gentamicin (GEN), ceftazidime (CAZ), and ciprofloxacin (CIP) against multidrug-resistant *Klebsiella pneumoniae* (Kpn) and *Klebsiella oxytoca* (Koxy).

Furthermore, similar results were observed for *K. oxytoca*. For GEN, MICs differed significantly (H = 21.3508, *p* = 0.0002698) when essential oils of oregano (*p* = 0.01355), summer savory (*p* = 0.04671), or thyme (*p* = 0.00001647) were added. For CAZ, a significant difference was identified using the Kruskal–Wallis test (H = 22.7266, *p* = 0.0001436) when carvacrol (*p* = 0.03063), oregano (*p* = 0.01331), summer savory (*p* = 0.01271), or thyme (*p* = 0.00002007) was added. Finally, for CIP, a significant difference was observed (H = 23.768, *p* = 0.0008886) when oregano (*p* = 0.0001976), summer savory (*p* = 0.003546), or thyme (*p* = 0.000002448) was added.

Although thyme contains the lowest carvacrol concentration, its activity does not always differ from that of other EOs, suggesting that its biological activity depends on the whole phytochemical matrix, not carvacrol alone. The modified checkerboard assay demonstrated that sub-inhibitory concentrations of carvacrol and the evaluated essential oils substantially enhanced the antibacterial activity of GEN, CIP, and CAZ against multidrug-resistant *Klebsiella* spp. The results indicated that although all compounds affect antibiotics, the essential oils achieved a greater reduction in antibiotic concentration than carvacrol alone. Furthermore, this result supports the hypothesis that carvacrol alone may not fully account for the enhanced antibacterial activity of complete essential oils, which may involve contributions from other phytochemical constituents or potential interactions among them.

### 3.4 *In vivo* toxicity of carvacrol and essential oils in invertebrate models

Finally, to evaluate the hypothesis that isolated compounds are less toxic than complete essential oils, the *in vivo* toxicity of carvacrol was evaluated and compared with that of the essential oils in *T. molitor* and *G. mellonella* larvae. An exploratory health index scoring system was used to assess larval health (Table 1), enabling semiquantitative evaluation of overall health status and detection of changes in larval activity, cocoon formation, melanization, and survival. Larval scores were recorded at 24 and 48 h after inoculation (Figure 5). We found that summer savory EO was lethal to *T. molitor* larvae, whereas the survival index for *G. mellonella* was 93%. Although toxicity was similar between carvacrol and oregano oil in the *T. molitor* model, there was no difference in melanization compared with the other treatments, suggesting that the humoral response was not activated *in vivo*, as supported by the effect observed in *G. mellonella*. The absence of detectable melanization suggests that immune activation may not be the primary mechanism underlying the compounds’ potential toxicity. Oregano essential oil, thyme essential oil, and carvacrol exhibited low toxicity in both *G*. *mellonella* and *T*. *molitor* under the experimental conditions evaluated, supporting their further investigation as potential antimicrobial adjuvants. However, invertebrate toxicity models provide only preliminary information regarding biological safety and cannot be extrapolated directly to mammalian systems. In summer savory, a toxic effect was observed, although further analysis is needed to determine whether it is dose-dependent or specific to the *T. molitor* model as no toxic effect was observed in *G. mellonella*. Additional immunological analyses would be required to confirm this hypothesis. The exploratory semiquantitative health assessment provided additional information regarding larval physiological status beyond survival alone.

**FIGURE 5:**
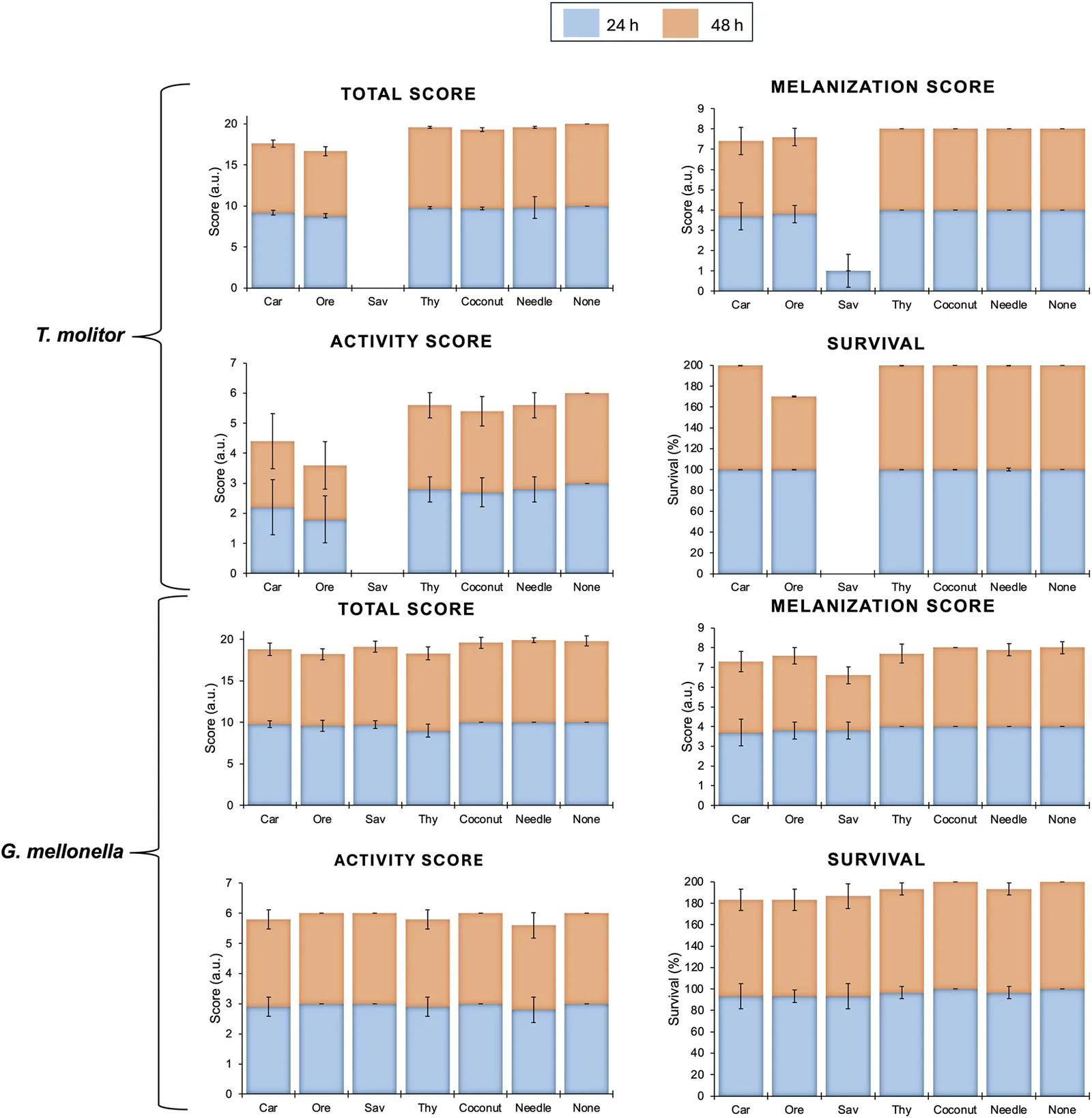
Toxicity assessment of carvacrol and essential oils in *Galleria mellonella* and *Tenebrio molitor* larvae after administration of carvacrol (Car), oregano essential oil (Ore), summer savory essential oil (Sav), and thyme essential oil (Thy) at concentrations corresponding to 2× MIC. Larvae were monitored daily for 24 h (blue bars) and 48 h (orange bars) after compound inoculation. Controls included coconut oil (vehicle), injection control (needle), and no-treatment control (none). Score values were assigned as indicated in Table 1.

## 4 Discussion

*Klebsiella pneumoniae* and *K. oxytoca* are opportunistic pathogens of increasing clinical concern due to multidrug resistance, particularly in pediatric settings. We evaluated the antibacterial activity of nine compounds and compared their efficacy with complete EOs rich in these constituents. Among the evaluated compounds, carvacrol showed the strongest antibacterial activity against clinical MDR strains, with inhibition zones >15 mm and bacteriostatic/bactericidal effects, yielding MIC values of 1,084.34 mg/mL for *K. pneumoniae* and 302.96 mg/mL for *K. oxytoca*.

The present study shows that although carvacrol is the most potent antibacterial compound among the evaluated phytochemicals, it does not fully replicate the antimicrobial efficacy of whole essential oils against clinical MDR *K. pneumoniae* and *K. oxytoca* isolates. If carvacrol were the main driver of antibacterial activity, its MIC (359.6 mg/mL for *K. pneumoniae* and 32.7 mg/mL for *K. oxytoca*) would be comparable to its concentration in the essential oils, especially oregano oil (79.7 mg/mL for *K. pneumoniae* and 8.6 mg/mL for *K. oxytoca*), where carvacrol is the main component. The difference in activity between isolated carvacrol and complete EOs suggests that other constituents contribute to the oils’ overall antibacterial efficacy, potentially through interactions with carvacrol. This finding highlights a critical limitation of the reductionist “single compound” approach and underscores the importance of the phytochemical composition of whole extracts for strong antimicrobial activity. Because all biological assays were performed using the same chemically characterized batch of each essential oil, we did not evaluate the influence of batch-to-batch variation or differences in phytochemical composition. Instead, the comparison between isolated carvacrol and the complete essential oils was intended to determine whether the biological activity of the whole phytochemical mixture differed from that of its major constituent.

Carvacrol exhibited strong antibacterial activity, as evidenced by inhibition zones >15 mm and by its bacteriostatic and bactericidal effects. These results align with previous reports describing carvacrol as a membrane-active compound that disrupts bacterial integrity, alters permeability, and interferes with cellular homeostasis (Asadi et al., 2023; Kashi et al., 2025). Moreover, its known ability to inhibit quorum sensing and biofilm formation likely contributes to its effectiveness against *Klebsiella*, a pathogen for which biofilm formation is a key virulence factor. However, despite its potency, carvacrol alone required higher concentrations to achieve bactericidal effects than the essential oils, indicating that additional components enhance antimicrobial efficacy.

A key finding of this study is that essential oils, particularly oregano and summer savory, exhibited superior bactericidal activity at lower concentrations than carvacrol alone. These findings suggest that phytochemical interactions may contribute to antibacterial efficacy beyond carvacrol alone. Compounds such as γ-terpinene, p-cymene, and thymol, present in varying proportions in these oils, may contribute to membrane destabilization, facilitate active compound penetration, or target complementary bacterial pathways. Interestingly, thyme essential oil contained only 1.83% carvacrol but 34.8% thymol, suggesting that antibacterial activity may also be driven by alternative phenolic monoterpenes. Thymol has been reported to disrupt bacterial membranes through mechanisms similar to those of carvacrol, which may partially explain the observed biological activity despite the markedly lower carvacrol content. Thymol is also present in oregano oil (1.26%) and summer savory oil (0.14%). An important finding of this study is that antibacterial efficacy did not correlate directly with the concentration of carvacrol in the essential oils. Based on their chemical composition, oregano and summer savory oils contained substantially higher levels of carvacrol than thyme oil; however, the observed biological activities could not be explained solely by the abundance of this compound. In particular, thyme essential oil retained antibacterial activity despite containing only 1.83% carvacrol. These observations indicate that the antimicrobial properties of essential oils arise from the combined contribution of multiple constituents rather than from the action of a single dominant molecule. More studies are needed to identify the components that confer activity on the essential oils as a whole.

The synergistic interactions observed between carvacrol/essential oils and conventional antibiotics (ceftazidime, gentamicin, and ciprofloxacin) are particularly relevant in the context of antimicrobial resistance. Remarkably, most combinations produced FICI values well below the conventional synergy threshold (≤0.5), with several yielding values below 0.1. Such low FICI values indicate that the combined antimicrobial effect exceeded the activity expected from either agent alone, suggesting a substantial increase in bacterial susceptibility following exposure to the natural products. Although checkerboard assays are the accepted standard for initial synergy screening, FICI values should be interpreted cautiously as the index is highly sensitive to pronounced reductions in the MICs of one or both compounds and may overestimate the magnitude of synergy under static experimental conditions. Nevertheless, the synergistic interactions observed in this study were reproducible across independent biological replicates and consistently detected against both *K. pneumoniae* and *K. oxytoca*, supporting their biological relevance rather than suggesting experimental artifacts. This positions carvacrol and essential oils as promising adjuvants in combination therapies to restore antibiotic efficacy against MDR pathogens. The observed synergistic interactions may result from membrane permeabilization caused by phenolic terpenes, which favor antibiotic uptake and reduce the effective concentration required to inhibit bacterial growth. The modified checkerboard assay demonstrated that sub-inhibitory concentrations of carvacrol and the evaluated essential oils can potentiate the activity of conventional antibiotics against *Klebsiella* spp. The observed reductions in antibiotic MICs suggest that these natural products may enhance bacterial susceptibility to antibiotics when used at concentrations below their individual inhibitory thresholds. However, the modified assay employed a fixed concentration of the natural product (1/4 MIC) combined with an antibiotic concentration gradient and therefore does not provide the reciprocal concentration–response matrix required for formal assessment of pharmacological synergy. Thyme essential oil demonstrated the strongest synergistic interactions against *K. pneumoniae*, despite containing only 1.83% carvacrol. This finding reinforces the hypothesis that antibiotic potentiation depends on the entire phytochemical composition rather than on carvacrol concentration alone. Unlike the other combinations, thyme combined with ciprofloxacin against *K. oxytoca* produced only an additive interaction. This species-specific response may reflect intrinsic differences in membrane composition, permeability, efflux activity, or fluoroquinolone resistance mechanisms that limit the essential oil’s potentiating effect. These findings suggest that the antibacterial activity of whole essential oils cannot be explained solely by the activity of carvacrol and are consistent with the hypothesis that interactions among multiple phytochemical constituents contribute to their biological efficacy.

*In vivo* toxicity assays using *G. mellonella* and *T. molitor* provide additional insight into the therapeutic potential of these compounds. Evaluating toxicity in *G*. *mellonella* and *T*. *molitor* offers complementary information on the acute toxicity of the evaluated natural products before progressing to vertebrate models. Both insects are widely accepted alternative *in vivo* models because they are inexpensive, ethically advantageous, and possess an innate immune system that shares several functional characteristics with the mammalian innate immune response, including cellular phagocytosis, antimicrobial peptide production, and melanization. Consequently, these models are increasingly used in the early stages of antimicrobial and natural product development. The absence of significant toxicity in carvacrol, oregano, and thyme oils supports their low toxicity, whereas the observed lethality of summer savory in *T. molitor* suggests species-specific or dose-dependent toxicity. Notably, the lack of a melanization response in *T. molitor* may indicate that the immune response is not strongly activated, which could reflect low immunogenicity or alternative modes of toxicity. Interestingly, although oregano essential oil contained the highest concentration of carvacrol, it exhibited minimal toxicity, whereas summer savory, with a lower carvacrol content, produced toxicity in *T. molitor*. Likewise, thyme essential oil, which contained only 1.83% carvacrol but high levels of thymol, also showed low toxicity. These observations indicate that toxicity cannot be explained solely by the concentration of a single compound. Instead, interactions among major and minor constituents are likely to modulate the overall biological response, emphasizing that both efficacy and safety depend on the composition of the complete phytochemical matrix. Consequently, the favorable toxicity profiles observed in this study should be interpreted as preliminary evidence supporting further preclinical investigation rather than as confirmation of clinical safety.

Using two independent insect models strengthens the present study as toxicity differed between *G. mellonella* and *T. molitor*. These discrepancies likely reflect species-specific differences in cuticle permeability, xenobiotic metabolism, immune responses, and detoxification pathways rather than inconsistencies in the biological activity of the evaluated compounds. Consequently, combining complementary invertebrate models may provide a more comprehensive early assessment of toxicity than relying on a single insect species. The low mortality and limited changes in larval health observed in the *G. mellonella* and *T. molitor* models provide preliminary evidence of low acute toxicity for the evaluated essential oils and carvacrol under the experimental conditions tested. These findings support further investigation of these compounds as potential antimicrobial agents or antibiotic adjuvants. However, invertebrate models provide an initial assessment of toxicity and cannot establish safety in mammals or humans. Further studies using mammalian cell models and appropriate preclinical toxicological approaches are required to determine their safety profile and therapeutic potential.

Collectively, our findings indicate that antibacterial efficacy arises not only from individual bioactive molecules but also from interactions across all phytochemical matrices. Consequently, strategies focused exclusively on isolating single compounds may overlook biologically relevant activities present in whole botanical extracts.

One limitation of the study was the small number of strains included in the analysis. We recognize that the number of clinical isolates evaluated was limited. Therefore, statistical analyses were interpreted cautiously and were intended to support descriptive comparisons rather than to establish definitive population-level estimates. Future studies with larger collections of genetically diverse clinical isolates will be necessary to confirm the observed trends. To ensure consistency, the essential oils used in the study were from the same lot across all experiments, so the carvacrol concentration remained consistent. Many studies have shown that the composition of essential oils can vary by season. Overall, this study demonstrates that the antibacterial activity of essential oils against MDR *Klebsiella* is not solely dependent on their components but rather on synergistic interactions within their complex phytochemical matrix. This has important implications for drug development as isolating single compounds may result in a loss of efficacy. Instead, preserving or rationally reconstructing phytochemical combinations could provide more effective antimicrobial strategies. Another limitation we reported is that, although phytochemical interactions are suggested, the specific compounds responsible for the enhanced antibacterial activity were not experimentally identified. Finally, we evaluated antibacterial activity exclusively against planktonic bacterial cells. Since biofilm formation is a major contributor to the persistence and antimicrobial tolerance of *Klebsiella* spp., future studies should investigate whether the essential oils evaluated here also inhibit biofilm formation, disrupt established biofilms, or enhance antibiotic activity against sessile bacterial populations.

Whole essential oils consistently outperformed isolated carvacrol, indicating that their antibacterial efficacy cannot be explained solely by the concentration or activity of this major constituent and highlighting the potential importance of the extracts’ phytochemical composition. The activity of thyme essential oil, despite its relatively low carvacrol content and high thymol abundance, further supports the contribution of multiple constituents to the overall antibacterial effects of whole essential oils. However, the present experimental design does not establish whether these constituents interact synergistically, additively, or independently. Definitive characterization of such interactions will require systematic evaluation of purified compounds, including carvacrol, thymol, p-cymene, and γ-terpinene, using defined combination assays and appropriate interaction models, such as checkerboard and isobologram analyses. Future studies should also investigate the molecular mechanisms underlying these potential interactions, optimize antibiotic combination strategies, and validate the observed effects in mammalian infection models. Standardization of essential oil composition will be important for evaluating reproducibility and advancing the potential translation of these findings toward clinical applications.

The present paradigm in natural product drug discovery often focuses on identifying and isolating a single bioactive molecule. However, our results indicate that this reductionist approach may overlook beneficial interactions that occur naturally within phytochemical matrices. Under the evaluated conditions, essential oils consistently showed greater antibacterial efficacy and stronger antibiotic-potentiating effects than carvacrol alone, even though carvacrol was their major active constituent. These findings suggest that preserving the complete phytochemical composition may be a more effective strategy for antimicrobial development than isolating individual compounds.

## Data Availability

The original contributions presented in the study are included in the article/Supplementary Material, further inquiries can be directed to the corresponding author.
