# Supplementary material for: Carvacrol exhibits potent anti-Klebsiella activity, whereas the multicomponent composition of essential oils may contribute to their enhanced antibacterial activity
Source: Front Pharmacol. 2026 Sep 18;17:1918941. doi: 10.3389/fphar.2026.1918941 (PMC13631094; doi:10.3389/fphar.2026.1918941)
Supplement: Supplementary file 1 [file Table1.docx]

Table S1. Composition of Summer Savory Essential Oil.

| Peak# | R.Time | Name | Area% |  |
| --- | --- | --- | --- | --- |
| 1 | 11.87 | alpha-Thujene | 1.29 |  |
| 2 | 12.26 | alpha-Pinene | 1.01 |  |
| 3 | 12.79 | Thujadiene | 0.02 |  |
| 4 | 13.14 | Camphene | 0.08 |  |
| 5 | 14.40 | Sabinene | 0.01 |  |
| 6 | 14.69 | beta-Pinene | 0.45 |  |
| 7 | 14.78 | 1-Octen-3-ol | 0.07 |  |
| 8 | 15.34 | Myrcene | 1.83 |  |
| 9 | 16.23 | delta-2-Carene | 0.02 |  |
| 10 | 16.37 | alpha-Phellandrene | 0.31 |  |
| 11 | 16.53 | delta-3-Carene | 0.07 |  |
| 12 | 17.05 | alpha-Terpinene | 3.47 |  |
| 13 | 17.54 | para-Cymene | 4.24 |  |
| 14 | 17.81 | Limonene | 0.32 |  |
| 15 | 17.90 | beta-Phellandrene | 0.21 |  |
| 16 | 17.98 | 1,8-Cineole | 0.02 |  |
| 17 | 18.21 | cis-beta-Ocimene | 0.01 |  |
| 18 | 18.90 | trans-beta-Ocimene | 0.10 |  |
| 19 | 19.85 | gamma-Terpinene | 33.49 |  |
| 20 | 20.45 | cis-Sabinene hydrate | 0.08 |  |
| 21 | 21.46 | Terpinolene | 0.09 |  |
| 22 | 21.78 | para-Cymenene | 0.02 |  |
| 23 | 22.50 | trans-Sabinene hydrate | 0.01 |  |
| 24 | 23.35 | 2,4-Dimethyl-2,4-heptadienal | 0.09 |  |
| 25 | 27.33 | Borneol | 0.04 |  |
| 26 | 27.94 | Terpinen-4-ol | 0.30 |  |
| 27 | 29.06 | alpha-Terpineol | 0.09 |  |
| 28 | 29.26 | cis-Dihydro carvone | 0.03 |  |
| 29 | 31.92 | Carvacrol methyl ether | 0.06 |  |
| 30 | 32.55 | Carvone | 0.02 |  |
| 31 | 34.87 | Isothymol | 0.02 |  |
| 32 | 35.48 | Thymol | 0.14 |  |
| 33 | 36.38 | Carvacrol | 48.83 |  |
| 34 | 40.31 | Carvacrol acetate | 0.17 |  |
| 35 | 41.23 | trans-beta-Damascenone | 0.01 |  |
| 36 | 43.85 | beta-Caryophyllene | 1.13 |  |
| 37 | 44.71 | trans-alpha-Bergamotene | 0.04 |  |
| 38 | 45.03 | Aromadendrene | 0.02 |  |
| 39 | 45.60 | Geranyl acetone | 0.01 |  |
| 40 | 45.95 | trans-beta-Farnesene | 0.02 |  |
| 41 | 46.07 | alpha-Humulene | 0.05 |  |
| 42 | 48.31 | Viridiflorene | 0.03 |  |
| 43 | 48.56 | Bicyclogermacrene | 0.01 |  |
| 44 | 49.13 | trans-trans-alpha-Farnesene | 0.03 |  |
| 45 | 49.35 | beta-Bisabolene | 1.43 |  |
| 46 | 50.28 | beta-Sesquiphellandrene | 0.02 |  |
| 47 | 51.28 | trans-alpha-Bisabolene | 0.13 |  |
| 48 | 53.40 | Spathulenol | 0.02 |  |
| 49 | 53.69 | Caryophyllene oxide | 0.05 |  |
| 50 | 67.91 | Phytone | 0.01 |  |
