# Supplementary material for: Carvacrol exhibits potent anti-Klebsiella activity, whereas the multicomponent composition of essential oils may contribute to their enhanced antibacterial activity
Source: Front Pharmacol. 2026 Sep 18;17:1918941. doi: 10.3389/fphar.2026.1918941 (PMC13631094; doi:10.3389/fphar.2026.1918941)
Supplement: Supplementary file 2 [file Table2.docx]

Table S2. Composition of Oregano Essential Oil.

| Peak# | R.Time | Name | Area% |
| --- | --- | --- | --- |
| 1 | 6.651 | Methyl alpha-methyl butyrate | 0.04 |
| 2 | 12.687 | Tricyclene | 0.01 |
| 3 | 12.839 | alpha-Thujene | 0.64 |
| 4 | 13.27 | alpha-Pinene | 0.65 |
| 5 | 13.822 | Thujadiene | 0.02 |
| 6 | 14.213 | Camphene | 0.34 |
| 7 | 15.858 | beta-Pinene+Octen-3-ol | 0.26 |
| 8 | 16.469 | Myrcene | 1.22 |
| 9 | 16.934 | 3-Octanol | 0.02 |
| 10 | 17.576 | alpha-Phellandrene | 0.19 |
| 11 | 17.725 | delta-3-Carene | 0.07 |
| 12 | 18.256 | alpha-Terpinene | 1.18 |
| 13 | 18.781 | para-Cymene | 4.9 |
| 14 | 19.06 | Limonene | 0.21 |
| 15 | 19.17 | beta-Phellandrene | 0.21 |
| 16 | 19.259 | 1,8-Cineole | 0.11 |
| 17 | 19.441 | cis-beta-Ocimene | 0.03 |
| 18 | 20.146 | trans-beta-Ocimene | 0.05 |
| 19 | 21.01 | gamma-Terpinene | 4.33 |
| 20 | 21.79 | cis-Sabinene hydrate | 0.27 |
| 21 | 22.819 | Terpinolene | 0.17 |
| 22 | 23.141 | para-Cymenene | 0.04 |
| 23 | 23.851 | Linalool | 4.72 |
| 24 | 24.027 | Hotrienol | 0.1 |
| 25 | 25.545 | cis-para-Menth-2-en-1-ol | 0.03 |
| 26 | 25.689 | alpha-Campholenal | 0.03 |
| 27 | 28.882 | Borneol | 1.19 |
| 28 | 29.469 | Terpinen-4-ol | 0.77 |
| 29 | 30.479 | trans-beta-Ocimenol | 0.05 |
| 30 | 30.631 | alpha-Terpineol | 0.11 |
| 31 | 33.427 | Carvacrol methyl ether | 0.12 |
| 32 | 34.188 | Carvone | 0.23 |
| 33 | 34.352 | Linalyl acetate | 0.09 |
| 34 | 35.505 | Carvenone | 0.03 |
| 35 | 36.988 | Thymol | 1.26 |
| 36 | 37.982 | Carvacrol | 71.47 |
| 37 | 38.692 | Methyl non-8-enoate | 0.17 |
| 38 | 39.213 | tert-Butyl-para-benzoquinone | 0.09 |
| 39 | 41.056 | Eugenol | 0.02 |
| 40 | 41.892 | Carvacryl acetate | 0.13 |
| 41 | 42.312 | 2-Allyl-para-cresol | 0.02 |
| 42 | 42.773 | Geranyl acetate | 0.02 |
| 43 | 43.22 | beta-Bourbonene | 0.03 |
| 44 | 44.179 | Methyl eugenol | 0.02 |
| 45 | 45.529 | beta-Caryophyllene | 1.31 |
| 46 | 46.332 | trans-alpha-Bergamotene | 0.03 |
| 47 | 46.589 | tert-Butylcatechol | 0.04 |
| 48 | 46.702 | Aromadendrene | 0.18 |
| 49 | 47.773 | alpha-Humulene | 0.06 |
| 50 | 48.964 | trans-Cadina-1(6),4-diene | 0.03 |
| 51 | 49.975 | Viridiflorene | 0.11 |
| 52 | 50.993 | beta-Bisabolene | 1.69 |
| 53 | 51.317 | gamma-Cadinene | 0.11 |
| 54 | 51.614 | delta-Cadinene | 0.07 |
| 55 | 51.935 | beta-Sesquiphellandrene | 0.03 |
| 56 | 52.92 | trans-alpha-Bisabolene | 0.03 |
| 57 | 53.52 | Thymohydroquinone | 0.15 |
| 58 | 55.107 | Spathulenol | 0.12 |
| 59 | 55.434 | Caryophyllene oxide | 0.22 |
| 60 | 58.865 | epi-alpha-Cadinol | 0.11 |
