# Supplementary material for: Carvacrol exhibits potent anti-Klebsiella activity, whereas the multicomponent composition of essential oils may contribute to their enhanced antibacterial activity
Source: Front Pharmacol. 2026 Sep 18;17:1918941. doi: 10.3389/fphar.2026.1918941 (PMC13631094; doi:10.3389/fphar.2026.1918941)
Supplement: Supplementary file 3 [file Table3.docx]

Table S3. Composition of Thyme Essential Oil. a. indicates the principal components of the oil. b. indicates carvacrol composition.

| Peak# | R.Time | Name | Area% |
| --- | --- | --- | --- |
| 1 | 6.22 | Methyl alpha-methyl butyrate | 0.56 |
| 2 | 8.707 | 3-cis-Hexenol | 0.04 |
| 3 | 11.784 | Tricyclene | 0.06 |
| 4 | 11.943 | alpha-Thujene | 1.97 |
| 5 | 12.342 | alpha-Pinene | 1.25 |
| 6 | 12.856 | Thujadiene | 0.03 |
| 7 | 13.23 | Camphene | 1.17 |
| 8 | 14.469 | Sabinene | 0.03 |
| 9 | 14.83 | Octen-3-ol | 1.32 |
| 10 | 15.139 | 3-Octanone | 0.07 |
| 11 | 15.412 | Myrcene | 2.22 |
| 12 | 15.828 | 3-Octanol | 0.13 |
| 13 | 16.444 | alpha-Phellandrene | 0.19 |
| 14 | 16.587 | delta-3-Carene | 0.12 |
| 15 | 17.113 | alpha-Terpinene | 1.88 |
| 16 | 17.726 | para-Cymene | 22.21 |
| 17 | 17.91 | Limonene | 0.56 |
| 18 | 18.007 | beta-Phellandrene | 0.21 |
| 19 | 18.089 | 1,8-Cineole | 0.31 |
| 20 | 18.952 | trans-beta-Ocimene | 0.05 |
| 21 | 19.861 | gamma-Terpinene | 13.72 |
| 22 | 20.54 | cis-Sabinene hydrate | 0.93 |
| 23 | 21.524 | Terpinolene | 0.12 |
| 24 | 21.828 | para-Cymenene | 0.04 |
| 25 | 22.54 | Linalool | 2.66 |
| 26 | 22.675 | trans-Sabinene hydrate | 0.11 |
| 27 | 25.726 | Camphor | 0.88 |
| 28 | 26.865 | Lavandulol | 0.02 |
| 29 | 27.47 | Borneol | 2.05 |
| 30 | 28.048 | Terpinen-4-ol | 1.03 |
| 31 | 28.518 | para-Cymen-8-ol | 0.04 |
| 32 | 29.138 | alpha-Terpineol | 0.12 |
| 33 | 29.357 | cis-Dihydro carvone | 0.03 |
| 34 | 31.358 | Thymol methyl ether | 0.14 |
| 35 | 32.001 | Carvacrol methyl ether | 1.2 |
| 36 | 33.08 | Geraniol | 0.1 |
| 37 | 34.895 | Isothymol | 0.2 |
| 38 | 35.812 | Thymol | 34.8 |
| 39 | 36.154 | Carvacrol | 1.83 |
| 40 | 39.124 | Thymol acetate | 0.08 |
| 41 | 39.497 | Eugenol | 0.05 |
| 42 | 41.127 | Isobornyl propanoate | 0.08 |
| 43 | 41.645 | beta-Bourbonene | 0.05 |
| 44 | 43.98 | beta-Caryophyllene | 3.65 |
| 45 | 44.975 | tert-Butylcatechol | 0.18 |
| 46 | 46.165 | alpha-Humulene | 0.13 |
| 47 | 46.433 | allo-Aromadendrene | 0.03 |
| 48 | 47.048 | Geranyl propionate | 0.09 |
| 49 | 47.384 | trans-Cadina-1(6),4-diene | 0.07 |
| 50 | 47.753 | Germacrene D | 0.1 |
| 51 | 48.649 | Bicyclogermacrene | 0.03 |
| 52 | 48.84 | alpha-Muurolene | 0.04 |
| 53 | 49.714 | gamma-Cadinene | 0.2 |
| 54 | 50.027 | delta-Cadinene | 0.19 |
| 55 | 52.285 | Geranyl butanoate | 0.01 |
| 56 | 53.463 | Spathulenol | 0.03 |
| 57 | 53.78 | Caryophyllene oxide | 0.42 |
| 58 | 55.719 | 1,10-Di-epi-cubenol | 0.02 |
| 59 | 57.224 | epi-alpha-Cadinol | 0.1 |
| 60 | 60.114 | Shyobunol | 0.05 |
