# Supplementary material for: Carvacrol exhibits potent anti-Klebsiella activity, whereas the multicomponent composition of essential oils may contribute to their enhanced antibacterial activity
Source: Front Pharmacol. 2026 Sep 18;17:1918941. doi: 10.3389/fphar.2026.1918941 (PMC13631094; doi:10.3389/fphar.2026.1918941)
Supplement: Supplementary file 4 [file Table4.docx]

Table S4. Strains included in this study.

| **Strain** | **Sample Origin** | **Inhibition Ratio (mm) / Interpretation** | | | | | | | | | | | | | | | | | |
| --- | --- | --- | --- | --- | --- | --- | --- | --- | --- | --- | --- | --- | --- | --- | --- | --- | --- | --- | --- |
|  |  | **CIP** | | **GEN** | | **CAZ** | | **TGC** | | **TET** | | **LNZ** | | **NFT** | | **DOX** | | **LEV** | |
| Kpn 1 | Blood | 7 | R | 7 | R | 7 | R | 11 | R | 10 | R | 7 | R | 7 | R | 15 | S | 35 | S |
| Kpn 2 | Urine | 7 | R | 7 | R | 7 | R | 11 | R | 9 | R | 7 | R | 7 | R | 7 | R | 36 | S |
| Kpn3 | Bronchial Aspirate | 7 | R | 7 | R | 7 | R | 14 | R | 10 | R | 9 | R | 9 | R | 9 | I | 36 | S |
| Koxy 1 | Blood | 7 | R | 13 | R | 12 | R | 11 | R | 20 | S | 7 | 7 | 7 | R | 20 | S | 30 | S |
| Koxy 2 | Urine | 7 | R | 7 | R | 7 | R | 10 | R | 30 | S | 7 | R | 7 | R | 20 | S | 36 | S |
| Koxy 3 | CSF | 7 | R | 10 | R | 19 | I | 12 | R | 14 | I | 7 | R | 7 | R | 18 | S | 30 | S |
| *Based on CLSI breakpoints, except for Tygecycline, when EUCAST value was considered. R:Resistant, I: Intermediate, S: Susceptible. CSF: Cerebrospinal Fluid. | | | | | | | | | | | | | | | | | | | |
